# Supplementary material for: Towards smart glasses for facial expression recognition using OMG and machine learning
Source: Sci Rep. 2023 Sep 25;13:16043. doi: 10.1038/s41598-023-43135-5 (PMC10520037; doi:10.1038/s41598-023-43135-5)
Supplement: Supplementary file 1 — Supplementary Information. [file 41598_2023_43135_MOESM1_ESM.docx]

## Supplementary Information

Table S. 1. Demographic information for each participant in the study.

| Participant ID | Age | Gender | Participant ID | Age | Gender |
| --- | --- | --- | --- | --- | --- |
| 1 | 25 | male | 15 | 26 | male |
| 2 | 23 | male | 16 | 23 | female |
| 3 | 25 | female | 17 | 41 | female |
| 4 | 21 | male | 18 | 34 | male |
| 5 | 25 | male | 19 | 47 | male |
| 6 | 27 | female | 20 | 18 | female |
| 7 | 24 | male | 21 | 20 | female |
| 8 | 25 | male | 22 | 17 | male |
| 9 | 30 | male | 23 | 30 | female |
| 10 | 22 | female | 24 | 17 | male |
| 11 | 30 | female | 25 | 33 | female |
| 12 | 23 | male | 26 | 42 | female |
| 13 | 23 | male | 27 | 17 | male |
| 14 | 22 | male |  |  |  |


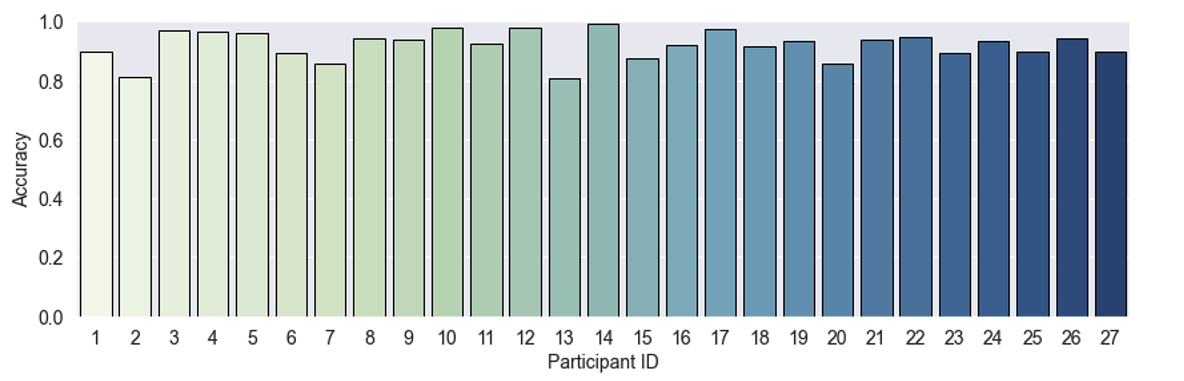


Figure S.1. Per-subject test accuracies from the LOSO evaluation presented in Figure 7 and Table 1 (section Machine learning for facial expression recognition from OCO™ sensors data).

| **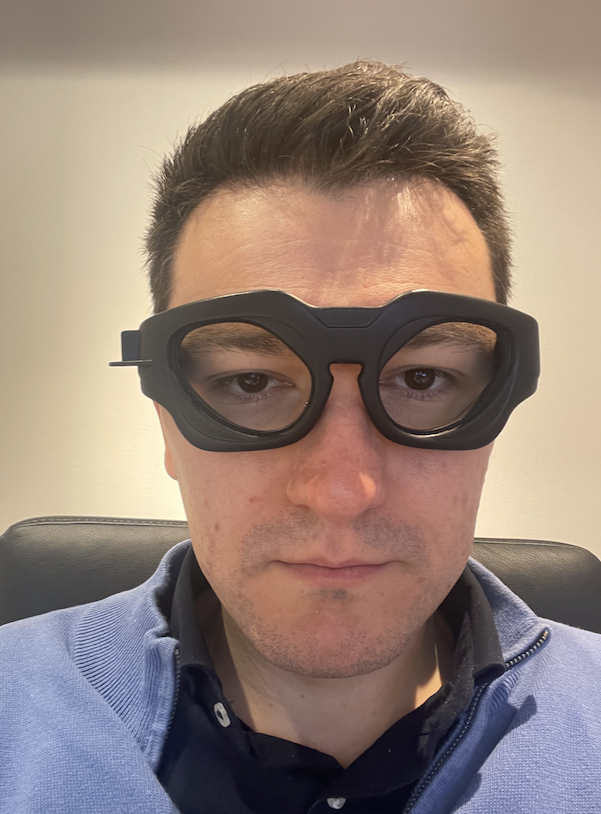 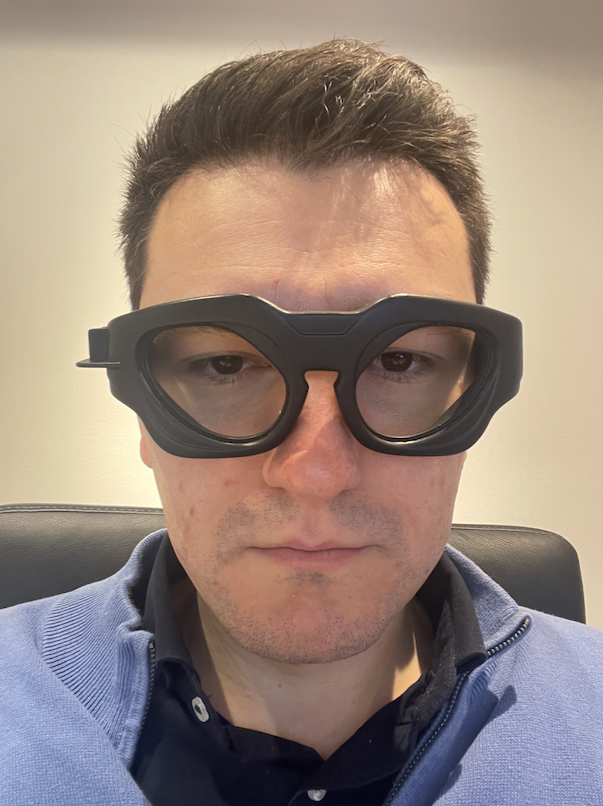 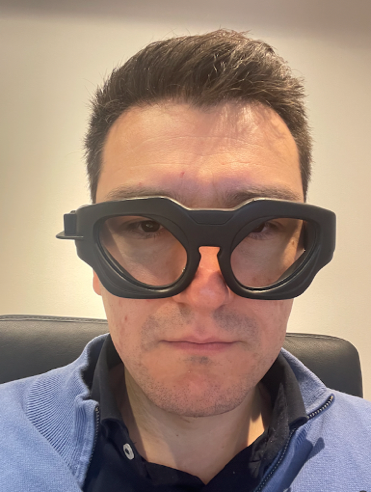** |
| --- |
| Figure S.2. From left to right: high, medium and low position of the glasses. |
